# Comparative utility of LC3, p62 and TDP-43 immunohistochemistry in differentiation of inclusion body myositis from polymyositis and related inflammatory myopathies

**DOI:** 10.1186/2051-5960-1-29

**Published:** 2013-07-01

**Authors:** Annie Hiniker, Brianne H Daniels, Han S Lee, Marta Margeta

**Affiliations:** 1 Department of Pathology, University of California San Francisco, San Francisco, CA 94143, USA; 2 Touro University College of Osteopathic Medicine, Vallejo, CA 94592, USA

**Keywords:** Inclusion body myositis, Polymyositis, COX-negative, LC3, p62, TDP-43

## Abstract

**Background:**

Inclusion body myositis (IBM) is a slowly progressive inflammatory myopathy of the elderly that does not show significant clinical improvement in response to steroid therapy. Distinguishing IBM from polymyositis (PM) is clinically important since PM is steroid-responsive; however, the two conditions can show substantial histologic overlap.

**Results:**

We performed quantitative immunohistochemistry for (1) autophagic markers LC3 and p62 and (2) protein aggregation marker TDP-43 in 53 subjects with pathologically diagnosed PM, IBM, and two intermediate T cell-mediated inflammatory myopathies (polymyositis with COX-negative fibers and possible IBM). The percentage of stained fibers was significantly higher in IBM than PM for all three immunostains, but the markers varied in sensitivity and specificity. In particular, both LC3 and p62 were sensitive markers of IBM, but the tradeoff between sensitivity and specificity was smaller (and diagnostic utility thus greater) for LC3 than for p62. In contrast, TDP-43 immunopositivity was highly specific for IBM, but the sensitivity of this test was low, with definitive staining present in just 67% of IBM cases.

**Conclusions:**

To differentiate IBM from PM, we thus recommend using a panel of LC3 and TDP-43 antibodies: the finding of <14% LC3-positive fibers helps exclude IBM, while >7% of TDP-43-positive fibers strongly supports a diagnosis of IBM. These data provide support for the hypothesis that disruption of autophagy and protein aggregation contribute to IBM pathogenesis.

## Background

Sporadic inclusion body myositis (IBM) is a common inflammatory myopathy that classically presents in older individuals with proximal lower extremity and finger flexor weakness; the clinical course is generally slow but progressive and unresponsive to immunosuppressive therapy, thus leading to significant disability. Histologically, IBM shows chronic myopathic changes in a background of T cell-rich endomysial inflammation [1-3]. In addition, IBM is marked by the presence of large protein aggregates associated with rimmed vacuoles (RVs) – cleared-out spaces surrounded by the basophilic granular material [1,3]. The pathogenesis of IBM is complex and not fully understood. Based on the histopathologic features of the disease, both degenerative and autoimmune etiologies have been proposed, but no one molecular mechanism has been broadly accepted [1,3-7].

The pathologic diagnosis of IBM requires its differentiation from two groups of disorders. First, IBM must be distinguished from other muscle disorders with RVs; these include hereditary inclusion body myopathies, some limb-girdle muscular dystrophies, many distal myopathies, and drug-induced autophagic vacuolar myopathies. Generally, the distinction between sporadic IBM and other myopathies with RVs is fairly straightforward: IBM shows a prominent T cell-rich endomysial inflammatory infiltrate and diffuse upregulation of major histocompatibility complex I (MHC-1) in muscle fibers; other myopathies with RVs do not [1]. Second, IBM must be distinguished from polymyositis (PM), another T cell-rich inflammatory myopathy with diffuse MHC-1 upregulation. Clinically, the distinction of PM from IBM is critical: the two diseases show markedly different response to corticosteroid therapy, with PM usually significantly responsive and IBM largely unresponsive to treatment. In contrast to PM, IBM generally shows chronic myopathic features (such as endomysial fibrosis and fiber size variation) and RVs [1,3]. However, standard histologic methods can visualize RVs only in the frozen tissue [1]; in addition, RVs are often scarce [3,8]. Thus, the current pathologic criteria for IBM are specific but not sensitive for diagnosis; in the absence of supporting clinical information, a significant fraction of IBM patients is misdiagnosed with chronic polymyositis, leading to unnecessary steroid treatment.

Given the limitations of the current diagnostic criteria, there has been substantial effort to identify an immunohistochemical marker for IBM with a higher sensitivity than RVs. Since protein aggregation is considered central to IBM pathogenesis, several groups have evaluated immunoreactivity for aggregation-prone proteins [including amyloid-β, amyloid-β precursor protein, phosphorylated neurofilament (SMI-31), ubiquitin, alpha-B crystallin, and TAR-DNA binding protein-43 (TDP-43)] in IBM specimens [9-13]. Many of these markers are specific but not sensitive for IBM and, like RVs, are limited by the scarcity of positive staining. For example, SMI-31 staining highlighted an average of 0.7% of fibers in IBM, less than the percentage of fibers with RVs [13]. Currently, the most promising protein aggregation marker is TDP-43: TDP-43 immunohistochemistry was positive in 21 of 27 cases (78%) of sporadic IBM in an initial study [14] and 77% of cases in a further study [12]. Overall, quantitation of TDP-43 has been variable and ranges from an average of <1% to 23% muscle fibers in IBM cases [10,15,16].

A different approach to finding a sensitive marker for IBM is to look for markers related to removal of abnormally aggregated proteins. In both animal [17] and human studies [18-20], RV formation has been linked to the impairment of autophagy, a catabolic process that targets cytoplasmic organelles and protein aggregates for lysosomal degradation [21]. Autophagy impairment leads to accumulation of autophagosomes, which can be detected by immunohistochemistry for autophagy proteins LC3 (microtubule-associated protein 1 light chain 3) and p62/SQSTM1; we have recently shown that immunostaining for either LC3 or p62 can replace electron microscopy in the diagnosis of drug-induced autophagic vacuolar myopathies [18,22]. LC3 and p62 have also been evaluated as markers of IBM. Temiz et al. have reported that LC3 aggregates are present in most cases of IBM and COX-negative polymyositis (PM-COX) and absent in most cases of PM; however, a threshold for deeming a case positive for each marker was not specified [12]. A second study found that more fibers were positive for p62 than for TDP-43 in IBM, but did not examine PM or other entities in the differential diagnosis [15]. The most complete study thus far evaluated p62 and TDP-43 staining in IBM, possible IBM (pIBM), and a small group (seven cases) of either PM or dermatomyositis (DM) [10]. An apparent difference in the mean fraction of p62- and TDP-43-positive fibers was demonstrated between IBM and PM/DM; however, statistical analysis was not performed, and DM is usually not in the pathologic differential diagnosis with IBM.

Though useful, these studies thus lack two critical elements. First, there has been no systematic quantification of all three stains across the same set of specimens, which would allow a better understanding of the role of autophagy impairment and protein aggregation in IBM pathogenesis. Second, almost none of these reports focused on the situation we consider most diagnostically challenging; namely, distinguishing IBM from PM in cases with atypical clinical history or incompletely developed histologic features. In the current study, we therefore undertake a quantitative and systematic study of LC3, p62, and TDP-43 immunohistochemistry in a broad spectrum of T-cell mediated inflammatory myopathies. We begin by establishing the sensitivity and specificity of each marker in the clear-cut cases of PM and IBM, then use these findings to evaluate more challenging cases with intermediate PM-COX and pIBM pathology.

## Methods

### Ethics statement

Study design was reviewed and approved by the University of California San Francisco (UCSF) Committee on Human Research (CHR). Given the non-invasive nature of the study and a minimal potential for harm to study participants, the informed consent requirement was waived by the CHR. No individually identifiable patient data is presented in this report.

### Objectives

The objective of this study was to determine (1) whether quantitative immunohistochemistry for LC3, p62, and/or TDP-43 can be used as a diagnostic tool to differentiate IBM from PM and (2) whether these markers can help classify intermediate forms of T cell-rich inflammatory myopathies (PM-COX and pIBM; further defined in the Participants section).

### Participants

To identify cases for the study, we performed a computerized search of the UCSF neuropathology case database spanning the interval between 1990 and 2012. Candidate cases (for which archival formalin-fixed, paraffin-embedded [FFPE] tissue was available) were classified into four groups (PM, PM-COX, pIBM, and IBM) by consensus of two Board-certified neuromuscular pathologists (HSL and MM), who were blinded to the clinical history and previous diagnoses. The classification was made after review of all available original light microscopy slides; these generally included hematoxylin and eosin (H&E) stain of the FFPE material; H&E, modified trichrome, ATPase (pH 9.2), NADH reductase, SDH, and COX stains of the frozen material; MHC-1 immunoperoxidase stain of the frozen material; and CD3, CD20 and CD8 immunoperoxidase stains of either the FFPE or frozen material. (All stains were not available in all cases, and the minimal stain set considered sufficient for classification included frozen section H&E [for all cases] and COX stain [for PM and PM-COX cases].) The diagnostic criteria used for classification are summarized in Table 1; cases with mixed features (for example, dermatomyositis/PM or dermatomyositis/IBM) and cases with unusual morphologic findings (for example, the presence of large lymphoid follicle-like inflammatory aggregates) were excluded. Clinical information available for each case was reviewed following initial histopathology classification, and a subset of cases was excluded from further study due to the presence of a concurrent disease (such as connective tissue disorder, lymphoproliferative neoplasm, or AIDS) that raised the possibility of secondary rather than primary inflammatory myopathy. Because approximately two thirds of our muscle biopsies come from outside referring institutions and are accompanied by limited clinical information, the clinical features were otherwise not incorporated into diagnostic criteria. Given that group assignment was based solely on morphologic features, no attempt was made to match participants by age, sex, or other demographic variables.

**Table 1:** Summary of consensus diagnostic criteria used for case classification

**Diagnostic criterion**	**PM**	**PM-COX**	**pIBM**	**IBM**
Lymphocytic endomysial inflammation^a^	Present	Present	Present	Present
Inflammatory infiltrate composition^b^	T-cell rich, B-cell poor	T-cell rich, B-cell poor	T-cell rich, B-cell poor	T-cell rich, B-cell poor
Degenerating / regenerating fibers^a^	Present (random distribution)	Present (random distribution)	Present (random distribution)	Present (random distribution)
Fiber invasion^b^	Present	Present	Present	Present
Diffuse MHC-1 positivity^b^	Present	Present	Present	Present
Endomysial fibrosis^a^	None or mild	None or mild	Moderate to severe	Moderate to severe
Fiber size variation^a^	None or mild	None or mild	Moderate to severe^c^	Moderate to severe^c^
Percentage of COX-negative fibers^a^	<1%	≥1%	Any (generally >1%)	Any (generally >1%)
Ragged red fibers^b^	Absent	Present	Either (generally present)	Either (generally present)
Classic rimmed vacuoles^a^	Absent	Absent	Absent	Present
Rimmed cracks or basophilic granular debris^b^	Absent	Absent	Present^d^	Either (generally present)

^a^ Main criteria (required for diagnosis / classification).

^b^ Supporting criteria.

^c^ Specimens with “moderate to severe fiber size variation” included the entire range of muscle fiber sizes (from atrophic to hypertropic; <5 to >100 μm) rather than just two populations of normal-sized and atrophic fibers, as can be seen in PM.

^d^ For pIBM classification, presence of rimmed cracks (incomplete rimmed vacuoles; Figure 5E, white arrowhead) or basophilic granular debris (material reminiscent of basophilic RV rim) was required in cases with moderate fibrosis but optional in cases with severe fibrosis.

### Procedures

#### Immunohistochemistry

Immunoperoxidase staining for LC3 (mouse monoclonal antibody, clone 5F10, Nanotools; 1:100 dilution following antigen retrieval) and p62/SQSTM1 (guinea pig polyclonal antibody, Progen Biotechnik; 1:100 dilution following antigen retrieval) was performed on FFPE tissue samples using Ventana Benchmark XT automated slide preparation system at the UCSF Brain Tumor Research Center Tissue Core as described previously [18]. Immunoperoxidase staining for TDP-43 (rabbit polyclonal antibody, Proteintech Group, Chicago, IL) was performed on FFPE tissue samples either manually (1:3000 antibody dilution; no antigen retrieval) or using a Leica Bond automated slide preparation system (1:1000 antibody dilution; antigen retrieval for 20 min in a buffer with pH = 6.0 and at a temperature of up to 100°C); the two staining methods produced essentially identical results.

#### Quantification

Quantification was performed on immunostained FFPE sections using a bright-field light microscope as described previously [18], with the investigator blinded to group assignment of each subject. Briefly, muscle fibers containing inclusions, RVs, threads/skeins (in case of TDP-43), or coarse sarcoplasmic puncta were counted as positive, while fibers lacking such staining were counted as negative. A total of 200 fibers/slide was counted in specimens with abundant positivity, while a total of 600 fibers/slide was counted in specimens with scarce or patchy positivity (to reduce the sampling error).

#### Imaging

Images were taken with a DP72 digital camera on a BX41 bright-field light microscope using cellSens Entry 1.6 software (all by Olympus) and were edited with Adobe Photoshop CS5 Version 12.1.32.

### Statistical methods

Data were analyzed with GraphPad Prism 6 statistical software. For between-group comparison of demographic variables, we used one-way ANOVA with post-hoc Tukey test (age) or chi-square test (sex). LC3, p62, and TDP-43 immunopositivity data showed significantly different variances among groups; thus, between-group comparison was performed by *t*-test with Welch’s correction or Kruskal-Wallis one-way ANOVA on ranks. To calculate diagnostic threshold values with optimal sensitivity and specificity, receiver operating characteristic (ROC) analysis was performed on the data from PM and IBM groups. All tests were two-tailed with α=0.05.

## Results

### Demographics

Participant assignment to study groups was based solely on morphologic criteria (see Methods and Table 1 for details) and no attempt was made to match participants by age, sex, or other demographic variables. As expected based on the previous reports, the average age of PM subjects (55.8 ± 12.0 y [mean ± SD]) was significantly lower than the age of IBM subjects (68.9 ± 7.1 y; p<0.05) but not significantly different from the ages of PM-COX subjects (64.4 ± 9.7 y) or pIBM subjects (59.6 ± 14.1 y). In addition, there was no significant difference in the average age between PM-COX, pIBM, and IBM groups. Sex distribution was not statistically different between the four study groups (17% [PM] vs. 62% [PM-COX] vs. 63% [pIBM] vs. 50% [IBM] female; p = 0.07).

The level of plasma creatine kinase (CK) prior to biopsy was available in the clinical record of 44 of 53 subjects (Table 1). Given the incompleteness of the data and variations in the reporting precision, statistical analysis of this parameter was not possible; however, CK > 1000 U/L was present in the majority of subjects in the PM group (10 of 12), half of subjects in the pIBM group (7 of 14), and the minority of subjects in the other two groups (1 of 9 subjects in the IBM group and 3 of 9 subjects in the PM-COX group). The demographic and CK level data for all subjects are shown in Table 2.

**Table 2:** Study subject characteristics

**Subject ID**	**Group**	**CK level (U/L)**	**Sex**	**Age at diagnosis**	**Rimmed vacuoles (%F)**	**LC3 (%FS)**	**p62 (%FS)**	**TDP-43 (%FS)**
1	PM	1,092	M	59	0.0	5.7	5.5	1.2
2	PM	“1,000 s”	M	85	0.0	6.0	10.0	0.5
3	PM	2,405	F	51	0.0	4.0	0.3	5.5
4	PM	1231	M	65	0.0	2.5	3.7	1.2
5	PM	1,497	M	47	0.0	1.7	11.0	0.2
6	PM	10,000	M	42	0.0	11.3	10.2	0.2
7	PM	477	F	66	0.0	3.8	4.0	0.5
8	PM	6,000 to 19,000	M	50	0.0	4.0	7.5	1.2
9	PM	3,000	M	59	0.0	6.7	8.3	0.8
10	PM	834	M	53	0.0	2.7	19.2	0.3
11	PM	2,300	M	43	0.0	2.7	4.0	0.2
12	PM	1,000 to 4,000	M	50	0.0	2.0	2.8	0.2
13	IBM	515 ( 1 y before biopsy)	F	64	2.7	31.0	18.5	8.5
14	IBM	5,201	F	75	1.2	19.5	18.0	1.0
15^a^	IBM	700	F	57	1.0	24.0	20.0	12.5
16^a^	IBM	444	M	62	4.3	29.5	59.5	40.5
17	IBM	547	M	77	2.5	15.5	16.5	9.0
18	IBM	NA	F	74	1.5	16.0	9.2	2.8
19	IBM	235 (5 y before biopsy)	F	73	3.0	34.0	32.5	16.0
20^a^	IBM	576 to 732	F	69	4.5	48.5	43.5	27.5
21	IBM	NA	M	75	3.0	11.0	22.5	18.5
22	IBM	900	M	59	2.2	33.5	36.0	16.0
23^a^	IBM	700	M	66	0.7	9.0	15.0	1.8
24	IBM	NA	M	76	0.5	32.5	12.0	0.2
25	PM-COX	NA	M	54	0.0	2.7	4.8	1.3
26	PM-COX	121	F	71	0.0	3.7	7.7	4.7
27	PM-COX	802	F	55	0.0	6.0	6.3	0.2
28	PM-COX	1,254 to 2,093	F	85	0.0	10.0	4.7	0.5
29^a^	PM-COX	395	M	75	0.0	13.7	13.8	1.0
30	PM-COX	174 to 229	M	54	0.0	3.8	8.0	1.0
31	PM-COX	NA	F	76	0.0	4.3	8.8	4.2
32	PM-COX	2,000 to 3,000	F	58	0.0	3.3	1.5	0.7
33^a^	PM-COX	NA	M	59	0.0	15.5	22.5	16.0
34	PM-COX	NA	F	63	0.0	16.5	28.5	4.5
35^a^	PM-COX	871	M	64	0.0	47.5	30.5	0.5
36	PM-COX	74,600	F	58	0.0	2.3	2.3	0.2
37	PM-COX	339 to 789	F	65	0.0	4.7	3.0	2.5
38	pIBM	6,500	F	42	0.0	3.8	12.8	1.2
39	pIBM	6,000 to 12,000	F	58	0.0	33.0	32.0	0.5
40	pIBM	802	F	71	0.0	5.3	2.5	0.3
41	pIBM	7,090	F	55	0.0	16.5	34.5	0.7
42	pIBM	74,000	F	61	0.0	18.5	10.5	4.2
43^a^	pIBM	871	M	62	0.0	25.5	10.5	6.5
44	pIBM	4,580	F	30	0.0	74.0	58.5	1.3
45	pIBM	NA	F	75	0.0	15.5	5.5	0.5
46^a^	pIBM	840	M	64	0.0	17.0	21.0	12.0
47	pIBM	10,000	M	53	0.0	16.0	15.3	1.2
48	pIBM	864	F	44	0.0	6.7	4.5	0.0
49	pIBM	600	F	83	0.0	24.7	2.5	0.3
50	pIBM	293	M	68	0.0	7.3	4.8	1.3
51	pIBM	6,000	F	50	0.0	21.5	17.0	0.8
52^a^	pIBM	222 (4 y after biopsy)	M	79	0.0	17.5	22.5	3.3
53	pIBM	NA	M	58	0.0	7.2	2.5	2.5

^a^ Classic IBM history.

*NA* not available, *%FS* the percentage of fibers staining, *%F* the percentage of fibers with rimmed vacuoles.

### Classic PM and IBM

**Figure 1:**
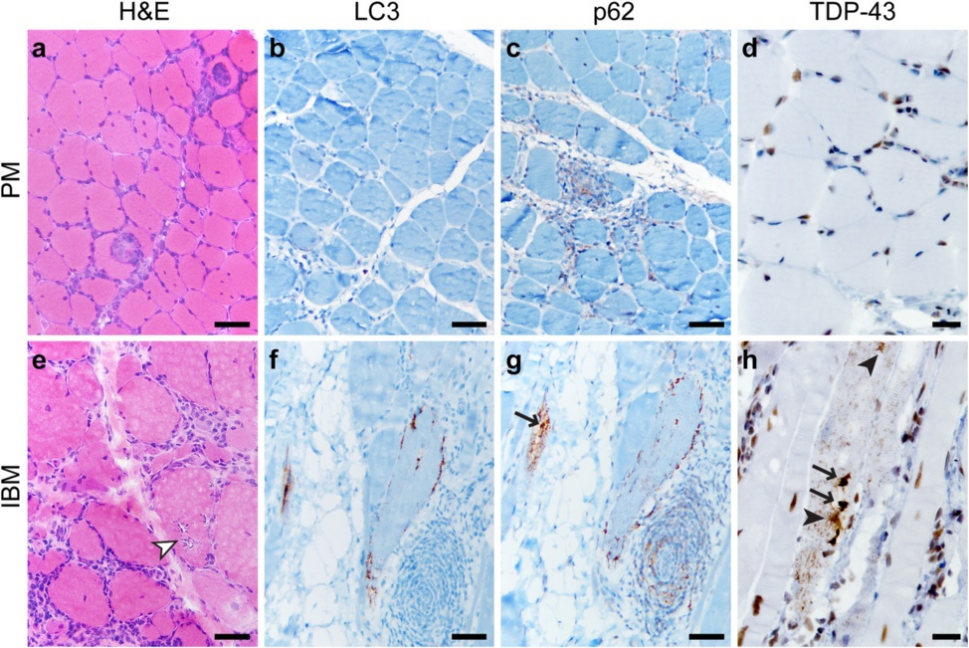
**PM and IBM staining patterns.** A representative case of PM (**a-d**; subject #10) shows endomysial lymphocytic inflammation and muscle fiber invasion but no chronic myopathic features (**a**; H&E stain of the frozen material). There is no significant sarcoplasmic staining with LC3 **(b)**, p62 **(c)**, or TDP-43 **(d)**, but TDP-43 stain highlights a subset of myofiber and inflammatory cell nuclei (internal positive control), while p62 faintly stains a subset of lymphocytes. A representative case of IBM (**e**-**h**, subject #22) shows endomysial inflammation accompanied by moderate to severe endomysial fibrosis, muscle fiber size variation, and RVs (white arrowhead) (**e**; H&E, frozen material). Staining for LC3 **(f)** and p62 **(g)** highlights RV rims; p62 also labels RV-associated protein aggregates (arrow) and scattered lymphocytes. TDP-43 immunostain **(h)** labels sarcoplasmic threads/skeins (black arrowheads), large protein aggregates (arrows), and coarse background puncta. Scale bars, 50 μM for **a-c** and **e-g**; 20 μM for **d** and **h**.

Immunohistochemistry for LC3, p62 and TDP-43 was performed on FFPE tissue. (While antibodies can show different antigen sensitivity on frozen and FFPE sections, we have previously demonstrated that LC3 and p62 antibodies used in this study perform similarly in both preparations [18].) In PM samples, there was little or no sarcoplasmic staining with any of the three antibodies (Figures 1b-d; subject #10); however, p62 faintly stained inflammatory cells (Figure 1c), while TDP-43 labeled the majority of myofiber and lymphocyte nuclei (Figure 1d). In some PM samples, rare fibers were LC3 and/or p62-positive; when present, such staining was generally coarsely punctate, with no labeling of large protein aggregates or RVs (not shown). (Normal human skeletal muscle shows no sarcoplasmic LC3 or p62 immunopositivity [18].) In contrast, IBM samples contained many LC3- and/or p62-positive fibers, typically showing coarse granularity and/or staining of RV rims (Figures 1f-g; subject #22); in addition, p62 often labeled sarcoplasmic protein aggregates and inflammatory cells (Figure 1g). TDP-43 showed three patterns of sarcoplasmic staining in IBM specimens (Figure 1h): large protein inclusions/aggregates (arrows, Figure 1h), thread-like skeins (black arrowheads; Figure 1h), and coarse granularity (seen in the background of the fiber with other inclusions); the rim of RVs typically was not labeled, and background nuclear staining was generally preserved.

**Figure 2:**
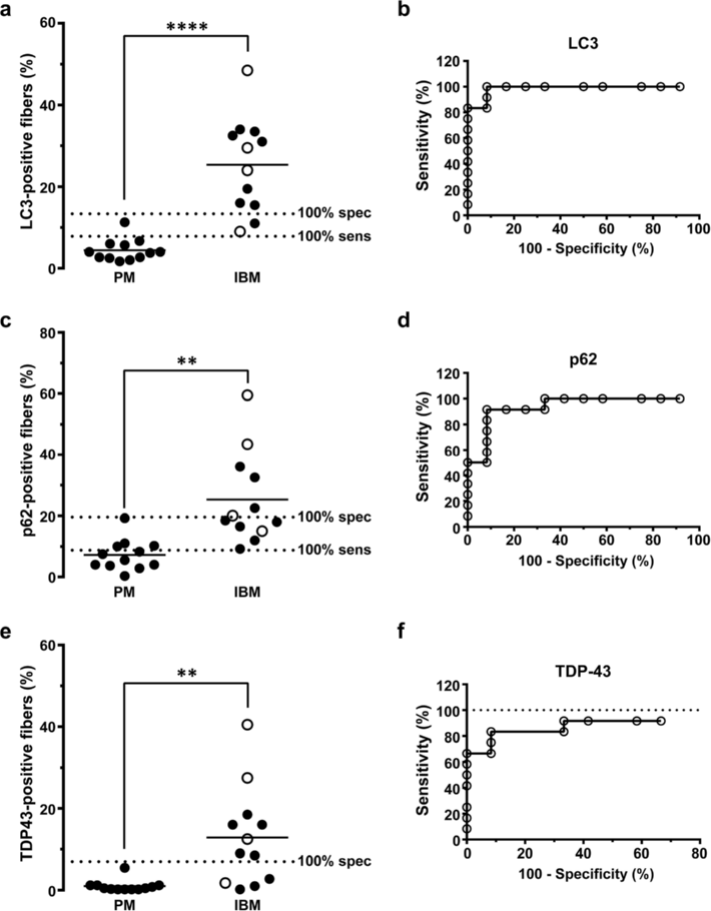
**Quantification of LC3, p62, and TDP-43 positive fibers in the PM and IBM groups.** The percentage of LC3- **(a)**, p62- **(c)**, and TDP-43-positive fibers **(e)** was significantly higher in the IBM group than the PM group. Each subject is represented with a symbol; the open symbols indicate subjects with known IBM clinical presentation. The unbroken lines designate group means, while dotted lines mark 100% sensitivity and 100% specificity cutoffs for each marker derived from ROC analysis. ****, p < 0.0001; **, p < 0.01. ROC analysis **(b, d,** and **f)** shows that quantitative immunohistochemistry for each of the three markers successfully differentiates IBM from PM subjects (p ≤ 0.001), although with varying tradeoffs between specificity and sensitivity. Of the three markers examined, only TDP-43 **(f)** failed to reach 100% sensitivity threshold, indicated by the dotted line in **(f)**.

To statistically compare the degree of LC3-, p62- and TDP-43-positivity between PM and IBM groups, we quantified the percentage of fibers staining (%FS) on each section. Data for individual subjects are shown in Table 2; interestingly, the percentage of LC3- and p62-positive fibers exceeded the percentage of fibers with RVs in all IBM cases, while TDP-43 data were more variable. The percentage of LC3-positive fibers was significantly higher in the IBM group (25.3 ± 3.3%FS) than in the PM group (4.4 ± 0.8%FS) (mean ± SEM, p<0.0001; two-tailed *t*-test with Welch’s correction; Figure 2a). Similar results were seen with p62 (IBM, 25.3 ± 4.3%FS; PM, 7.2 ± 1.5%FS; mean ± SEM, p=0.001; Figure 2c) and TDP-43 immunohistochemistry (IBM, 12.9 ± 3.5%FS; PM, 1.0 ± 0.4%FS; mean ± SEM, p=0.006; Figure 2e). ROC analysis showed that while all three immunohistochemical tests effectively distinguished IBM from PM specimens (p ≤ 0.001 for area under the ROC curve), there were large differences in sensitivity and specificity for each diagnostic marker (Figures 2b, 2d and 2f). In particular, both LC3 and p62 were sensitive markers of IBM, but the tradeoff between sensitivity and specificity was smaller for LC3 (100% specificity and 83% sensitivity for IBM using a threshold value of 13.4%FS) than for p62 (100% specificity and 50% sensitivity for IBM using a threshold value of 19.6%FS). TDP-43 immunopositivity was a highly specific marker of IBM, but the sensitivity of this test was low: a threshold value of 7%FS excluded all PM cases but captured only 67% (8 of 12) of IBM cases. In fact, the sensitivity of TDP-43 immunohistochemistry did not reach 100% even when the threshold value was set at the very low value of 0.3%FS (Figure 2f). Complete ROC analysis data are shown in Additional file 1: Table S1 (LC3), Additional file 2: Table S2 (p62), and Additional file 3: Table S3 (TDP-43).

Four of 12 subjects in the IBM group (and no subjects in the PM group) had clinical presentation classic for IBM (weakness of quadriceps and finger flexor muscles and CK lower than 1000 U/L) and would thus be classified as “definite IBM” using the European Neuromuscular Center (ENMC) criteria [23]; these subjects are designated by superscript ^a^ in Table 1 and by empty symbols in Figure 2. (The other 8 subjects in the IBM group either had atypical clinical presentation or, more commonly, lacked sufficient clinical information to make definitive assessment either way.) Interestingly, LC3, p62 and TDP-43 immunopositivities were not uniformly high in muscle biopsies from the 4 subjects with classic IBM history; rather, they showed a large range of labeling that spanned the entire spectrum of %FS values seen in the IBM group, with 3 of 4 subjects meeting or exceeding the 100% specificity threshold set for each of the three markers (Figures 2a, 2c, and 2e).

Taken together, these data indicate that (1) all three immunohistochemical markers effectively distinguish the IBM subject group from the PM subject group; (2) LC3 immunohistochemistry shows the best tradeoff between sensitivity and specificity as a diagnostic test applied to *individual biopsies* to confirm (or exclude) the diagnosis of IBM; and (3) given the high specificity of TDP-43 immunohistochemistry for IBM, TDP-43 immunopositivity confers additional support for a diagnosis of IBM and can thus be helpful in cases that have atypical clinical presentation or lack adequate clinical information.

### PM-COX

**Figure 3:**
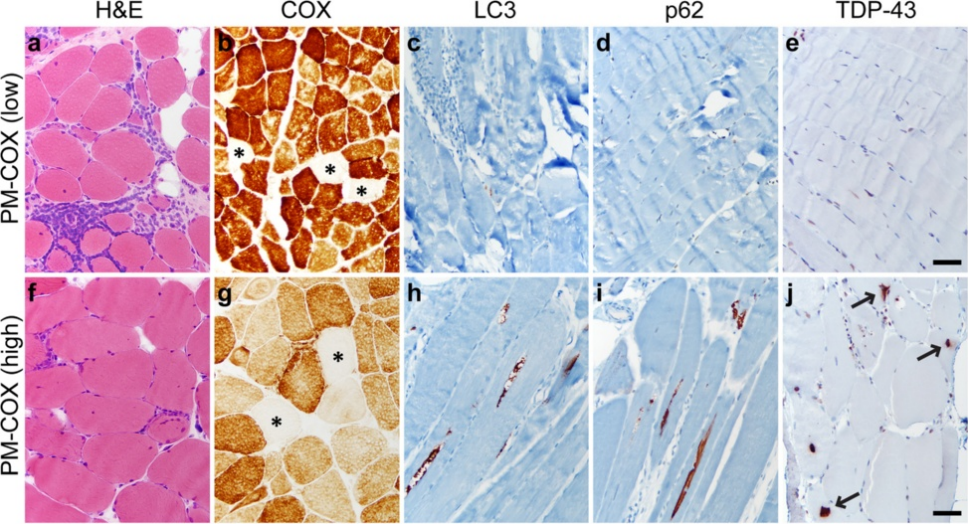
**PM-COX staining patterns.** PM-COX cases were histologically similar to classic PM, with endomysial inflammation, fiber invasion, and lack of well-developed chronic myopathic features (**a** and **f**; H&E, frozen material), but with ≥ 1% COX-negative fibers (**b** and **g**; COX stain, frozen material; COX-negative fibers are marked by asterisks). The majority of PM-COX samples (designated PM-COX low) showed no significant sarcoplasmic labeling for LC3 **(c)**, p62 **(d)**, or TDP-43 **(e)**. In a subset of samples (designated PM-COX high), LC3 labeled RV rims **(h)**, p62 labeled RV rims and small protein aggregates **(i)**, while TDP-43 labeled sarcoplasmic skeins and large protein aggregates (arrows; **j**). **a-e**, subject #37, **f-j**, subject #33; scale bar, 50 μM.

PM-COX shows worse response to steroid therapy than classic PM and is thus thought to represent either a form of progression from PM toward IBM or an early stage of IBM with incompletely developed pathologic features [8,12,24]. To evaluate whether immunohistochemistry for LC3, p62 and/or TDP-43 can distinguish PM-COX from classic PM and/or IBM, we evaluated 13 specimens with histologic features of polymyositis but ≥1% COX-negative fibers (for full diagnostic criteria, see Table 1). The majority of PM-COX specimens showed only minimal LC3, p62 or TDP-43 immunopositivity and thus resembled PM more than IBM; a representative biopsy from this subgroup (subject #37) is shown in Figures 3a-e. However, a few PM-COX specimens showed a significant degree of LC3-, p62-, and TDP-43 immunostaining, resembling classic IBM (see Table 2 for quantification); the single biopsy positive for all three markers (subject #33) is shown in Figures 3f-j.

**Figure 4:**
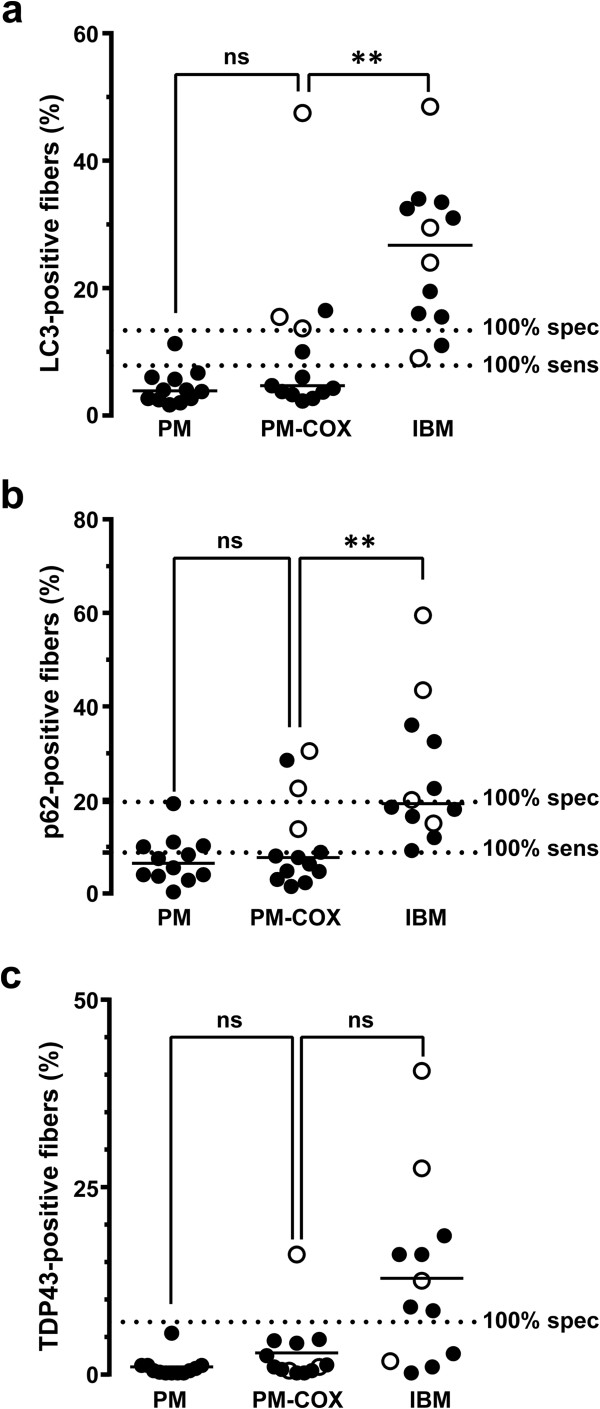
**Quantification of LC3, p62, and TDP-43 positive fibers in the PM-COX group.** The percentage of LC3- **(a)** and p62-positive fibers **(b)** was significantly lower in the PM-COX group than in the IBM group, but similar to the PM group. With TDP-43 **(c)**, there was no statistically significant difference between the PM-COX group and either the PM or IBM group. Each subject is represented with a symbol; the open symbols indicate subjects with known IBM clinical presentation. The unbroken lines designate group medians, while the dashed lines mark 100% sensitivity and 100% specificity cutoffs for each marker (derived from the ROC analysis shown in Figure 2). **, p < 0.01.

Quantitative comparison of the entire PM-COX group with the PM and IBM groups is shown in Figure 4. For all three markers, there was no statistically significant difference in %FS between the PM-COX group (median: LC3, 4.7%FS; p62, 7.7%FS; TDP43, 1.0%FS) and the PM group (median: LC3, 3.9%FS; p62, 6.5%FS; TDP43, 0.5%FS) (p>0.05, Kruskal-Wallis ANOVA on ranks). In contrast, the fraction of LC3- and p62-postive fibers (but not TDP-43-positive fibers) was significantly lower in the PM-COX group than in the IBM group (median: LC3, 26.8%FS; p62, 19.3%FS; TDP43, 10.8%FS) (p<0.01 for LC3 and p62, p>0.05 for TDP-43). Interestingly, there was no correlation between the fraction of ragged red and COX-negative fibers and the degree of LC3, p62, and TDP-43 immunopositivity (Additional file 4: Table S4).

Three of 13 subjects that showed PM-COX pathology had clinical presentations classic for IBM and would thus be classified as “probable IBM” using the ENMC criteria. (As with the IBM group, the other 10 subjects either had clinical presentation more consistent with PM or, more frequently, lacked sufficient clinical information to make definitive assessment either way.) Among the 3 subjects with IBM presentation (designated by open symbols in Figure 4), all 3 subjects exceeded the IBM 100% specificity threshold for LC3, 2 subjects exceeded the IBM 100% specificity threshold for p62, and 1 subject (#33, shown in Figures 3f-j) exceeded the IBM 100% specificity threshold for TDP-43. While the interpretation of this finding is limited by the low number of subjects with well-defined clinical history in our study set, these data suggest that, in cases with PM-COX histology, LC3 immunopositivity may identify the patients that are in the early stages of IBM and thus unlikely to respond to immunosuppressive therapy.

Taken together, the data indicate that the PM-COX group is heterogeneous but overall shows a low degree of LC3 and p62 immunopositivity more similar to classic PM than classic IBM. Nonetheless, a subset of PM-COX subjects with clinical presentation suggestive of IBM showed high labeling for autophagic markers LC3 and p62, but not for aggregation marker TDP-43; this finding supports the hypothesis that autophagy impairment occurs early and accumulation of misfolded proteins late in IBM pathogenesis [12,25].

### pIBM

**Figure 5:**
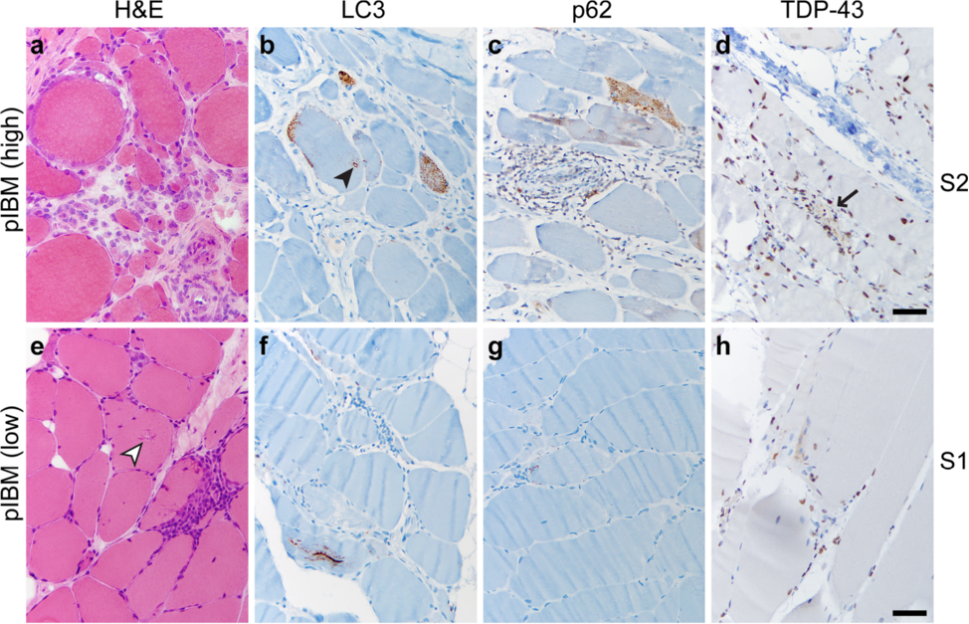
**pIBM staining patterns.** pIBM cases were histologically similar to classic IBM, with endomysial inflammation, fiber invasion, and moderate-severe chronic myopathic features, but without classic RVs (**a** and **f**; H&E, frozen material); “rimmed cracks” (white arrowhead in **e**) were present in a subset of specimens. The majority of pIBM samples (designated pIBM high) showed well-developed labeling for LC3 (**b**) and p62 (**c**); TDP-43 immunopositivity was less commonly observed (**d**; arrow marks a single TDP-43 positive fiber). In the example shown (subject #52), many fibers showed dense coarse puncta and rare RV-like structures (black arrowhead in **b**). In a smaller subset of samples (designated pIBM low), little or no labeling was seen with all three markers **(f-h)**. **a**-**d**, subject #52, **e**-**h**, subject #51; scale bar, 50 μM.

Current diagnostic criteria for IBM require identification of RVs (Figure 1e, white arrowhead) in the context of a chronic T-cell rich inflammatory myopathy; however, many biopsies lack RVs while meeting all other diagnostic criteria for IBM and are thus currently diagnosed as possible IBM (pIBM). [Sixteen specimens included in the pIBM group in our study lacked well-developed RVs but showed either severe chronic myopathic features or moderate chronic myopathic features together with basophilic granular debris and “rimmed cracks” (incompletely developed RVs lacking central clearing; Figure 5e, white arrowhead); for full diagnostic criteria, see Table 1.] Given that the lack of RVs in the majority of pIBM cases likely represents a sampling error rather than a true finding, we hypothesized that the fraction of fibers positive for autophagic markers LC3 and p62 (but not necessarily protein aggregation marker TDP-43) would be high in pIBM specimens. Indeed, most pIBM biopsies showed a high degree of LC3 and p62 immunopositivity and a low degree of TDP-43 immunopositivity; a representative example is shown in Figures 5a-d (subject #52). A subset of pIBM cases, however, was essentially immunonegative for all three markers examined; a representative example is shown in Figures 5e-h (subject #51). Interestingly, when pIBM subjects were stratified by CK level with a cutoff value of 1000 U/L, both low and high CK subgroups showed a similar range of labeling for LC3 and TDP-43; in contrast, p62 labeling was significantly lower in pIBM subjects with low CK level (Additional file 5: Figure S1).

**Figure 6:**
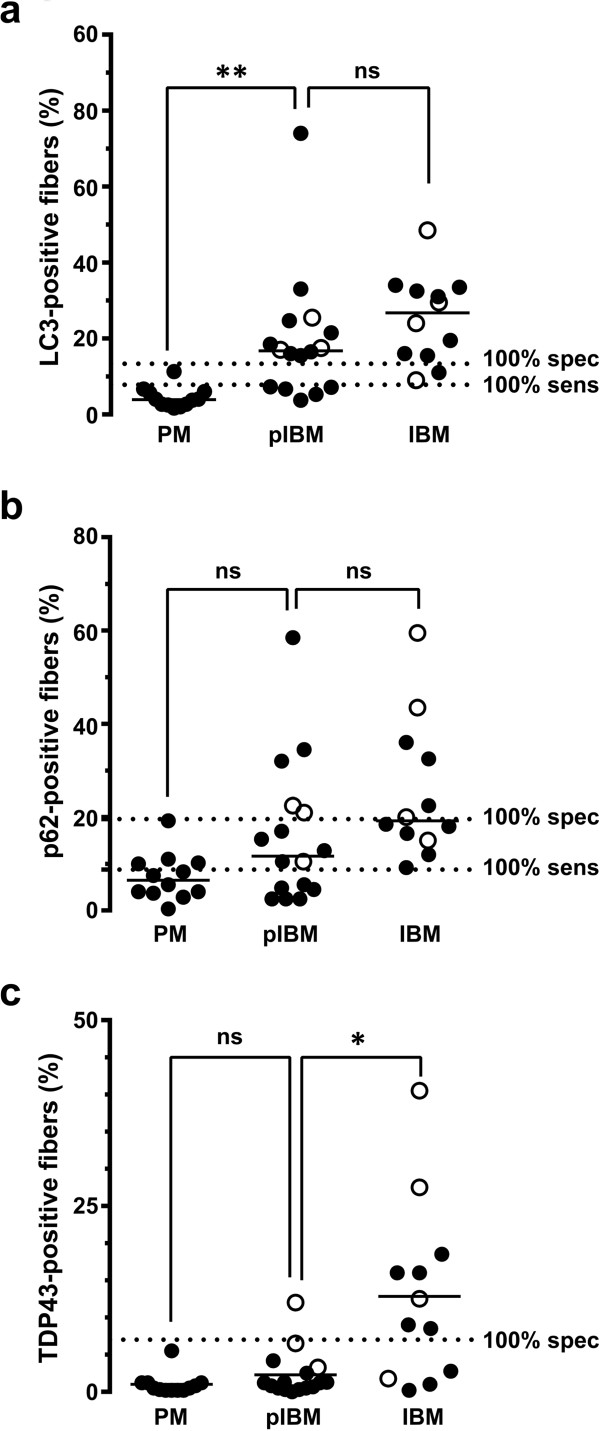
**Quantification of LC3, p62, and TDP-43 positive fibers in the pIBM group.** The percentage of LC3-positive fibers **(a)** was significantly higher in the pIBM group than in the PM group, but similar to the IBM group. With p62 **(b)**, there was no statistically significant difference between the pIBM group and either the PM or IBM group. The percentage of TDP-43-positive fibers **(c)** was significantly lower in the pIBM group than in the IBM group, but similar to the PM group. Each subject is represented with a symbol; the open symbols indicate subjects with known IBM clinical presentation. The unbroken lines designate group medians, while the dotted lines mark 100% sensitivity and 100% specificity cutoffs for each marker (derived from the ROC analysis shown in Figure 2). **, p < 0.01; *, p < 0.05.

Quantitative comparison of the entire pIBM group with the PM and IBM groups is shown in Figure 6. The fraction of LC3-positive fibers was significantly higher in the pIBM group (median, 16.8%FS) than in the PM group (median, 3.9%FS; p<0.01), but not significantly different from that observed in the IBM group (median, 26.8%FS; p>0.05, Kruskal-Wallis ANOVA on ranks). p62 immunohistochemistry showed a large spread of %FS values in the pIBM group (median, 11.7%FS), with no significant difference from either the PM group (median, 6.5%FS; p>0.05) or the IBM group (median, 19.3%FS; p>0.05). Finally, TDP-43 immunopositivity was low in all but one pIBM subject; thus, the fraction of TDP-43-immunopositive fibers in the pIBM group (median, 1.2%FS) was significantly lower than in the IBM group (median, 10.8%FS; p<0.05) and not significantly different from that observed in the PM group (median, 0.5%FS; p>0.05).

Three of 16 subjects in the pIBM group had clinical presentation classic for IBM and would thus be classified as “probable IBM” using the ENMC criteria. (As with the PM-COX and IBM groups, the other 13 subjects either had clinical presentation more consistent with PM or, more commonly, lacked sufficient clinical information to make definitive assessment either way.) Among the 3 pIBM subjects with IBM clinical history (designated by open symbols in Figure 6), all 3 subjects exceeded the IBM 100% specificity threshold for LC3, 2 subjects exceeded the IBM 100% specificity threshold for p62, and only 1 subject exceeded the IBM 100% specificity threshold for TDP-43. These results are reminiscent of those observed in the 3 PM-COX subjects with clinical features of IBM, again consistent with the notion that LC3 immunopositivity represents an early and TDP-43 immunopositivity a late marker of IBM.

## Discussion

IBM differs from other inflammatory myopathies by its lack of responsiveness to immunosuppressive therapy and poor prognosis. Thus, accuracy of pathologic diagnosis is critical, particularly when a patient is not clinically evaluated by a neuromuscular neurology specialist; however, differentiation of IBM from PM (which shows a significant histologic overlap) can be difficult. In this study, we quantitatively evaluated three immunohistochemical markers, LC3, p62 and TDP-43, for their diagnostic utility in differentiating IBM from PM and intermediate T cell-rich inflammatory myopathies, PM-COX and pIBM.

Several earlier studies have examined LC3, p62 or TDP-43 staining in the setting of IBM; however, no single work quantitatively compared all three markers on the same set of well-defined specimens. Of the three markers, TDP-43 has been the best studied: similar to our results, other authors have found that it stains most but not all cases of IBM [12,14,16]. Quantitative studies of TDP-43 staining (based on immunofluorescence or immunoperoxidase labeling of frozen sections) have been somewhat variable, with mean %FS in IBM ranging from <1% to 23% [10,15,16]; our results (based on immunoperoxidase labeling of FFPE sections) are very similar (mean %FS, 13%; 67% sensitivity using 7% labeling cutoff and 90% sensitivity using 1% labeling cutoff), indicating that the two approaches give comparable results. Quantitative studies of p62 and LC3 have been less frequent, and no study has quantitatively compared the two markers we found the most useful: LC3 and TDP-43.

In our study, LC3, p62, and TDP-43 all effectively distinguished the IBM subject group from the PM subject group; however, LC3 immunohistochemistry showed the best tradeoff between sensitivity and specificity for IBM as a diagnostic test applied to an individual case (100% specificity and 83% sensitivity for IBM using a threshold value of 13.4%FS; Figure 2a). p62 staining was qualitatively similar to LC3 staining, consistent with the idea that accumulation of either LC3-labeled autophagosomes or p62-positive aggregates can serve as a marker of autophagic flux inhibition [26]. However, p62 staining showed a larger tradeoff between sensitivity and specificity than LC3 staining (100% specificity and 50% sensitivity for IBM using a threshold value of 19.6%FS; Figure 2c). Interestingly, both LC3 and p62 labeled coarse sarcoplasmic puncta and the rim of RVs, suggesting that RVs are delimited by built-up unprocessed autophagosomes. The TDP-43 staining pattern, in contrast, was both qualitatively and quantitatively unique. Like LC3 and p62, TDP-43 staining was often positive in coarse sarcoplasmic puncta. Unlike LC3 and p62, however, TDP-43 labeled sarcoplasmic skeins and large protein aggregates (which were largely LC3- and p62-negative) and did not label the RV rim (which was typically LC3- and p62-positive). While TDP-43 staining was detectable in only 67% of our IBM cases (using 7%FS cutoff), it was highly specific for IBM in the setting of a T cell-rich inflammatory myopathy, with essentially no staining in 11 of 12 PM biopsies. (In contrast, TDP-43 immunohistochemistry is positive in other myopathies with RVs [14,27] and thus does not differentiate sporadic IBM from hereditary inclusion body myopathies.) Based on these findings, we therefore suggest that the most useful immunohistochemical approach to differentiate IBM from PM in a muscle biopsy is to use a panel of LC3 and TDP-43 antibodies: a cutoff of <14%FS LC3 helps rule out IBM, while >7%FS TDP-43 strongly supports a diagnosis of IBM. Because >7%FS TDP-43 is highly specific for IBM, TDP-43 immunohistochemistry can be particularly useful in cases with limited or atypical clinical history. Importantly, because we optimized all immunohistochemical stains for FFPE tissue, this method enables diagnosis of IBM even in situations when frozen tissue is not available.

To determine whether immunopositivity for LC3, p62 and/or TDP43 provides additional information in diagnostically challenging cases, we evaluated two intermediate T cell-rich inflammatory myopathies – PM-COX and pIBM. PM-COX has features of both PM (the absence of chronic myopathic features and RVs) and IBM (the presence of COX-negative fibers). This group, designated “PM-Mito” or “PM/IBM” in some earlier studies, was shown to respond less well to steroid therapy than classic PM [8,12,24]. Our PM-COX group showed a low%FS for LC3 and p62 (median of 4.7%FS and 7.7%FS, respectively) that was more similar to the PM group (median of 3.9%FS and 6.5%FS) than to the IBM group (median of 26.8%FS and 19.3%FS). This LC3 finding is in apparent contrast to the work of Temiz et al., who showed that 87% of their PM-Mito cases were positive for LC3 [12]. We reconcile this apparent discrepancy by noting that all of our PM-COX cases showed at least small amount of positivity for LC3; however, %FS LC3 for IBM was much greater. Since the findings of Temiz et al. are not quantitative, the two results may well be the same, highlighting the additional information obtained by quantifying %FS rather than using a binary system of positive versus negative staining. An alternative possibility is that all of the PM-Mito cases in the study by Temiz et al. had clinical features of IBM; our PM-COX cases that met “probable IBM” ENMC criteria also showed high degree of LC3 immunopositivity (open symbols in Figure 4a; see below for further discussion). Like PM-COX biopsies, pIBM biopsies had features of both PM (the absence of RVs) and IBM (the presence of chronic myopathic features), but were histologically closer to the IBM end of the spectrum. The pIBM group as a whole showed a high %FS for LC3 (median, 16.8%FS) and a low %FS for TDP-43 (median, 1.2%FS), consistent with the idea that the majority of pIBM cases represent an early stage of IBM with incompletely developed pathologic features.

A clear limitation of our work is that, due to our role as a neuropathology referral center, the clinical information was incomplete for the majority (~ 2/3) of our study subjects. However, a subset of biopsies from clinically well-worked up subjects with classic IBM presentation highlights a few trends that warrant further study. The four (of 12) subjects in the IBM group that met “definite IBM” ENMC criteria showed a large range of staining that spanned the entire spectrum of IBM %FS values, with 3 of 4 subjects meeting or exceeding the 100% specificity threshold set for each of the three markers. In addition, 3 (of 13) subjects with PM-COX pathology and 3 (of 16) subjects with pIBM pathology met “probable IBM” ENMC criteria; all 6 of these subjects (100%) met or exceeded the >14%FS of LC3 threshold set for sensitive diagnosis of IBM. While limited by small sample size, these data suggest that >14%FS LC3 immunopositivity might be useful as a cutoff value for patients that are unlikely to respond to immunosuppressive therapy.

One model of IBM pathogenesis suggests that cytoplasmic protein accumulation occurs in a stepwise fashion, with impairment of autophagic flux (detected through LC3 and p62 accumulation) occurring first and aggregation of TDP-43 and other misfolded proteins occurring later [12,25]. Collectively, our results are in agreement with this model; however, a conclusive evidence for this sequence of events would require a positive correlation between the length of symptoms and the degree of LC3- and TDP-43-immunopositivity or evidence of progression from LC3-only to combined LC3 and TDP-43-immunopositivity in serial biopsies from the same patients. The presence of ragged red and COX-negative fibers in both PM-COX and IBM biopsies is consistent with a possible involvement of impaired mitophagy (mitochondrial autophagy) in these diseases. However, the degree of LC3, p62, and TDP-43 staining did not correlate with the percentage of COX-negative or ragged red fibers in our PM-COX specimens (Additional file 4: Table S4), suggesting that mitochondrial abnormalities can precede autophagy impairment in the IBM progression. Thus, many questions regarding IBM pathogenesis, including the precise role of autophagy and inflammation as well as the apparent chronologic sequence of particular protein accumulation, remain to be answered.

### Strengths and limitations

The major strengths of the current study are (1) the focus on a differential diagnosis (PM-IBM spectrum of T cell-rich inflammatory myopathies) that is frequently encountered in everyday muscle pathology practice; (2) the use of well-defined pathologic criteria for PM and IBM; (3) the inclusion of two intermediate conditions, PM-COX and pIBM, with equally well defined pathologic criteria; (4) the quantitative study design, which enabled calculations of sensitivity and specificity values for different diagnostic thresholds, and (5) the evaluation of three different markers across the same specimen set, enabling direct comparison of their sensitivity and specificity for IBM diagnosis. The major limitations are (1) the incompleteness of clinical record for approximately two thirds of our subjects, whose biopsies were received from outside institutions by our referral practice; and (2) the lack of clinical follow-up information for the same subset of subjects.

## Conclusion

In summary, we showed that quantitative immunohistochemistries for autophagic marker LC3 and protein aggregation marker TDP-43 can be useful ancillary tools for pathologic differentiation of PM from IBM and possible IBM precursor conditions, PM-COX and pIBM. By reducing the number of cases with equivocal diagnosis, particularly in a common setting of limited clinical information and/or suboptimally processed specimen lacking the frozen tissue, the widespread use of these immunostains has the potential to reduce the number of patients receiving inappropriate treatment.

## Competing interest

The authors declare that they have no competing interests.

## Authors’ contributions

AH, HSL and MM designed research; AH performed initial case selection and retrieved clinical data from the pathology case database and electronic medical record; HSL and MM classified subjects into experimental groups based on blinded review of slides; BHD performed all quantifications and prepared data tables; AH and MM performed statistical analyses; MM acquired images and prepared figures; AH and MM wrote the paper; MM supervised the project. All authors read and approved the final manuscript.

## Supplementary Material

Additional file 1: Table S1 LC3 immunohistochemistry: sensitivity and specificity for IBM diagnosis at different cutoff values (from ROC analysis). Click here for file

Additional file 2: Table S2 p62 immunohistochemistry: sensitivity and specificity for IBM diagnosis at different cutoff values (from ROC analysis). Click here for file

Additional file 3: Table S3 TDP-43 immunohistochemistry: sensitivity and specificity for IBM diagnosis at different cutoff values (from ROC analysis). Click here for file

Additional file 4: Table S4 Percentage of COX-negative and ragged red fibers in PM-COX group; LC3, p62, and TDP-43 data for the same subjects are included for comparison. Click here for file

Additional file 5: Figure S1 Comparison of the degree of LC3, p62, and TDP-43 immunopositivity between pIBM subjects with the low and high CK level. 14 pIBM subjects with known CK level (Table 2) were stratified into low CK subgroup (CK ≤ 1000 U/L) and high CK subgroup (CK > 1000 U/L). The percentage of LC3- (**a**) and TDP-43-positive fibers (**c**) was not significantly different between the two pIBM subgroups, while the percentage of p62-positive fibers was significantly lower in the low CK pIBM subgroup than the high CK pIBM subgroup (**b**). Each subject is represented with a symbol; the open symbols indicate subjects with known IBM clinical presentation. The unbroken lines designate group means, while dotted lines mark 100% sensitivity and 100% specificity cutoffs for each marker derived from ROC analysis. *, p < 0.05. Click here for file
