# Supplementary material for: Comparative utility of LC3, p62 and TDP-43 immunohistochemistry in differentiation of inclusion body myositis from polymyositis and related inflammatory myopathies
Source: Acta Neuropathol Commun. 2013 Jul 1;1:29. doi: 10.1186/2051-5960-1-29 (PMC3893502; doi:10.1186/2051-5960-1-29)
Supplement: Additional file 2: Table S2 — p62 immunohistochemistry: sensitivity and specificity for IBM diagnosis at different cutoff values (from ROC analysis). [file 2051-5960-1-29-S2.doc]

| **Cutoff (%FS)** | **Sensitivity (%)** | **95% CI** | **Specificity (%)** | **95% CI** |
| --- | --- | --- | --- | --- |
| > 1.580 | 100 | 73.54% to 100.0% | 8.333 | 0.2108% to 38.48% |
| > 3.265 | 100 | 73.54% to 100.0% | 16.67 | 2.086% to 48.41% |
| > 3.850 | 100 | 73.54% to 100.0% | 25 | 5.486% to 57.19% |
| > 4.750 | 100 | 73.54% to 100.0% | 41.67 | 15.17% to 72.33% |
| > 6.500 | 100 | 73.54% to 100.0% | 50 | 21.09% to 78.91% |
| > 7.900 | 100 | 73.54% to 100.0% | 58.33 | 27.67% to 84.83% |
| > 8.750 | 100 | 73.54% to 100.0% | 66.67 | 34.89% to 90.08% |
| > 9.600 | 91.67 | 61.52% to 99.79% | 66.67 | 34.89% to 90.08% |
| > 10.10 | 91.67 | 61.52% to 99.79% | 75 | 42.81% to 94.51% |
| > 10.60 | 91.67 | 61.52% to 99.79% | 83.33 | 51.59% to 97.91% |
| > 11.50 | 91.67 | 61.52% to 99.79% | 91.67 | 61.52% to 99.79% |
| > 13.50 | 83.33 | 51.59% to 97.91% | 91.67 | 61.52% to 99.79% |
| > 15.75 | 75 | 42.81% to 94.51% | 91.67 | 61.52% to 99.79% |
| > 17.25 | 66.67 | 34.89% to 90.08% | 91.67 | 61.52% to 99.79% |
| > 18.25 | 58.33 | 27.67% to 84.83% | 91.67 | 61.52% to 99.79% |
| > 18.85 | 50 | 21.09% to 78.91% | 91.67 | 61.52% to 99.79% |
| > 19.60 | 50 | 21.09% to 78.91% | 100 | 73.54% to 100.0% |
| > 21.25 | 41.67 | 15.17% to 72.33% | 100 | 73.54% to 100.0% |
| > 27.50 | 33.33 | 9.925% to 65.11% | 100 | 73.54% to 100.0% |
| > 34.25 | 25 | 5.486% to 57.19% | 100 | 73.54% to 100.0% |
| > 39.75 | 16.67 | 2.086% to 48.41% | 100 | 73.54% to 100.0% |
| > 51.50 | 8.333 | 0.2108% to 38.48% | 100 | 73.54% to 100.0% |

**Table S2.** p62 immunohistochemistry: sensitivity and specificity for IBM diagnosis at different cutoff values (from ROC analysis).
