# Supplementary material for: Comparative utility of LC3, p62 and TDP-43 immunohistochemistry in differentiation of inclusion body myositis from polymyositis and related inflammatory myopathies
Source: Acta Neuropathol Commun. 2013 Jul 1;1:29. doi: 10.1186/2051-5960-1-29 (PMC3893502; doi:10.1186/2051-5960-1-29)
Supplement: Additional file 4: Table S4 — Percentage of COX-negative and ragged red fibers in PM-COX group; LC3, p62, and TDP-43 data for the same subjects are included for comparison. [file 2051-5960-1-29-S4.doc]

| **Case Number** | **COX-negative (%)** | **Ragged Red (%)** | **LC3 (%)** | **p62 (%)** | **TDP-43 (%)** |
| --- | --- | --- | --- | --- | --- |
| 25 | 1.8 | 1.3 | 2.7 | 4.8 | 1.3 |
| 26 | 15.5 | 3.3 | 3.7 | 7.7 | 4.7 |
| 27 | 3.3 | 3.2 | 6.0 | 6.3 | 0.2 |
| 28 | 1.5 | 3.3 | 10.0 | 4.7 | 0.5 |
| 29^a^ | 3.8 | 3.2 | 13.7 | 13.8 | 1.0 |
| 30 | 15.5 | 4.0 | 3.8 | 8.0 | 1.0 |
| 31 | 4.7 | 2.8 | 4.3 | 8.8 | 4.2 |
| 32 | 4.5 | 2.3 | 3.3 | 1.5 | 0.7 |
| 33^a^ | 5.7 | 2.5 | 15.5 | 22.5 | 16.0 |
| 34 | 1.7 | 2.2 | 16.5 | 28.5 | 4.5 |
| 35^a^ | 2.0 | 1.5 | 47.5 | 30.5 | 0.5 |
| 36 | 2.8 | 2.2 | 2.3 | 2.3 | 0.2 |
| 37 | 3.5 | 1.8 | 4.7 | 3.0 | 2.5 |

**Table S4.** Percentage of COX-negative and ragged red fibers in PM-COX group; LC3, p62, and TDP-43 data for the same subjects are included for comparison.

^a^ classic IBM history
