# Supplementary figures and images for: Comparative utility of LC3, p62 and TDP-43 immunohistochemistry in differentiation of inclusion body myositis from polymyositis and related inflammatory myopathies
Source: Acta Neuropathol Commun. 2013 Jul 1;1:29. doi: 10.1186/2051-5960-1-29 (PMC3893502; doi:10.1186/2051-5960-1-29)

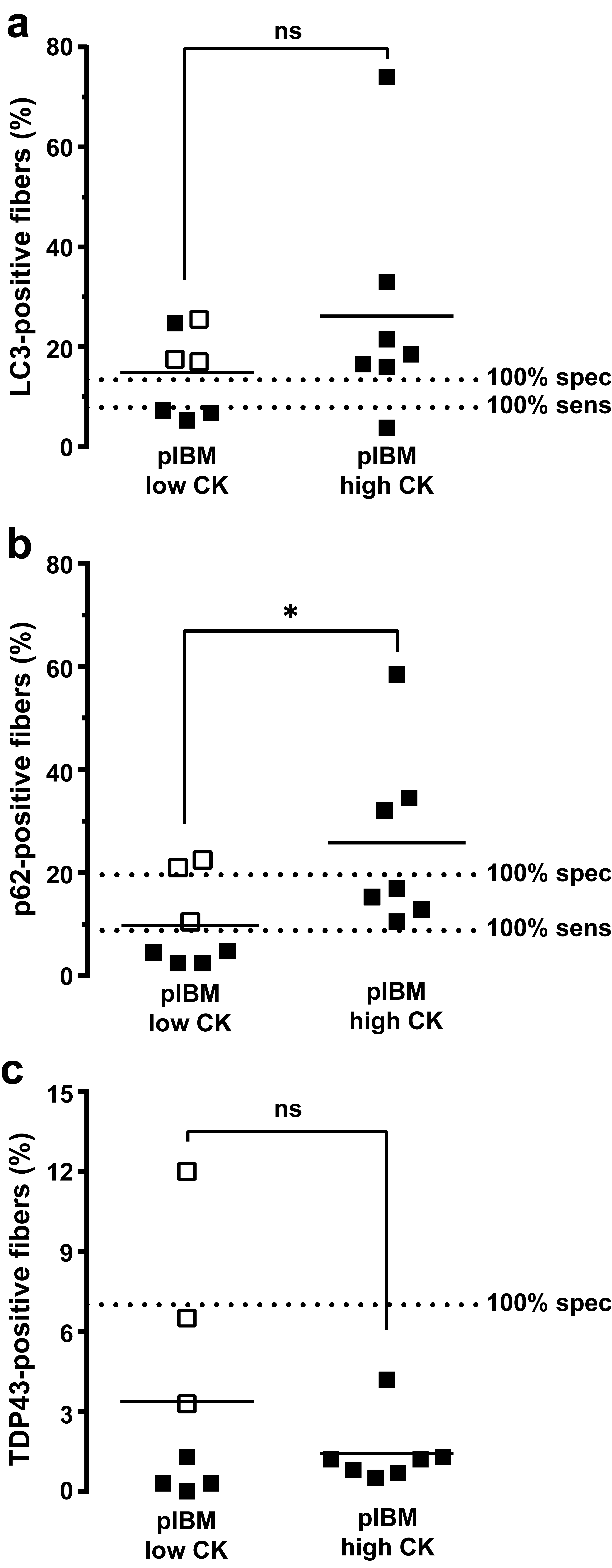

Supplement: Additional file 5: Figure S1 — Comparison of the degree of LC3, p62, and TDP-43 immunopositivity between pIBM subjects with the low and high CK level. 14 pIBM subjects with known CK level (Table 2) were stratified into low CK subgroup (CK ≤ 1000 U/L) and high CK subgroup (CK > 1000 U/L). The percentage of LC3- (a) and TDP-43-positive fibers (c) was not significantly different between the two pIBM subgroups, while the percentage of p62-positive fibers was significantly lower in the low CK pIBM subgroup than the high CK pIBM subgroup (b). Each subject is represented with a symbol; the open symbols indicate subjects with known IBM clinical presentation. The unbroken lines designate group means, while dotted lines mark 100% sensitivity and 100% specificity cutoffs for each marker derived from ROC analysis. *, p < 0.05. [file 2051-5960-1-29-S5.tiff]
